# Encephalomalacia/gliosis, deep venous thrombosis, and cancer in Arg393His antithrombin Hanoi and the potential impact of the β-amyloid precursor protein (APP) on thrombosis and cancer

**DOI:** 10.3934/Neuroscience.2022010

**Published:** 2022-04-21

**Authors:** Khue Vu Nguyen

**Affiliations:** Former Institution Attended:; 1 Department of Medicine, Biochemical Genetics and Metabolism, The Mitochondrial and Metabolic Disease Center, School of Medicine, University of California, San Diego, Building CTF, Room C-103, 214 Dickinson Street, San Diego, CA 92103-8467, USA; 2 Department of Pediatrics, University of California, San Diego, School of Medicine, San Diego, La Jolla, CA 92093-0830, USA

**Keywords:** Antithrombin (AT), Thrombosis, Encephalomalacia/gliosis, Cerebral venous thrombosis (CVT), Deep venous thrombosis (DVT), Venous thromboembolism (VTE), Pulmonary embolism (PE), Cancer, Kidney cancer, Warfarin, Low-molecular-weight heparin (LMWH), Tumor suppressor protein p53 (TP53), Human homologue of the murine double minute 2 protein (HDM2), Central nervous system (CNS), Hypoxanthine-guanine phosphoribosyltransferase (HGprt) enzyme, Hypoxanthine phosphoribosyltransferase 1 (*HPRT1*) gene, Lesch-Nyhan disease (LND), β-amyloid precursor protein (APP), APP-like protein-1 (APLP1), APP-like protein-2 (APLP2), Epigenetics, Epistasis, Alternative splicing, Antisense drugs

## Abstract

A heterozygous Arg393His point mutation at the reactive site of antithrombin (AT) gene causing thrombosis in a Vietnamese patient is reported and named as Arg393His in AT-Hanoi. The present variant is characterized by a severe reduction of functionally active AT plasma concentration to 42% of normal resulting in multiple severe thrombotic events such as cerebral venous thrombosis (CVT) (encephalomalacia/gliosis), recurrent deep venous thrombosis (DVT) and the development of kidney cancer. Today the complexity of thrombophilia has grown with appreciation that multiple inherited and acquired risk factors may interact to result in a clinically thrombotic phenotype. This article focuses on the following issues: (1) pathophysiology and clinical conditions of Arg393His in AT-Hanoi; (2) “two way association” between cancer and thrombosis in which venous thromboembolism (VTE) can be both a presenting sign and a complication of cancer; (3) efficacy of anticoagulants used for the prevention of cancer-related thrombosis; (4) conditions of acquired risk factors such as cancer or genetic disorders via epigenetic modifications in gene-gene (epistasis) and/or gene-environment interactions such as in Lesch-Nyhan disease (LND), in which the β-amyloid precursor protein (APP) that may interact to predispose a patient to thrombosis and cancer. It is also necessary to study the hypoxanthine-guanine phosphoribosyltransferase (HGprt) enzyme, AT, and APP using expression vectors for exploring their impact on LND, thrombosis as well as other human diseases, especially the ones related to APP such as Alzheimer's disease (AD) and cancer. For such a purpose, the construction of expression vectors for HGprt and APP, with or without the glycosyl-phosphatidylinositol (GPI) anchor, was performed as described in Ref. #148 (Nguyen, K. V., Naviaux, R. K., Nyhan, W. L. Lesch-Nyhan disease: I. Construction of expression vectors for hypoxanthine-guanine phosphoribosyltransferase (HGprt) enzyme and amyloid precursor protein (APP). *Nucleosides Nucleotides Nucleic Acids* 2020, 39: 905–922). In the same manner, the construction of expression vectors for AT and APP can be performed as shown in Figure 6. These expressions vectors, with or without GPI anchor, could be used as tools for (a) studying the effects of Arg393His mutation in AT; (b) studying the emerging role of Arg393His mutation in AT and cancer; (c) studying intermolecular interactions between APP and AT.

Furthermore, the construction of expression vectors as described in Ref. #148, especially the one with GPI, can be used as a model for the construction of expression vectors for any protein targeting to the cell plasma membrane for studying intermolecular interactions and could be therefore useful in the vaccines as well as antiviral drugs development (studying intermolecular interactions between the spike glycoprotein of the severe acute respiratory syndrome coronavirus 2, SARS-CoV-2, as well as its variants and the angiotensin-converting enzyme 2, ACE2, in coronavirus disease 2019 (COVID-19) [155],[156], for example).

## 1. Introduction

Coagulation is a normal physiological process by which blood changes the form from liquid to a gel, forming a blood clot. It potentially results in hemostasis, a process to prevent and stop bleeding, leading to the cessation of blood loss from a damaged vessel, followed by repair. Coagulation is highly conserved throughout biology. In all mammals, coagulation involves both a cellular (platelet) and a protein (coagulation factor) component. The coagulation system overlaps with the immune system. Coagulation can physically trap invading microbes in blood clots. Also, some products of the coagulation system can contribute to the innate immune system by their ability to increase vascular permeability and act as chemotactic agents for phagocytic cells. In addition, some of the products of the coagulation system are directly antimicrobial. For example, beta-lysine, an amino acid produced by platelets during coagulation, can cause lysis of many Gram-positive bacteria by acting as a cationic detergent. Disorders of coagulation are disease states, which can result in problems with hemorrhage, bruising, or thrombosis. There are many excellent reviews that summarize the coagulation process in detail but are otherwise beyond the scope of this article. Briefly, various substances are required for the proper functioning of the coagulation cascade including coagulation factors: factors essential to normal blood clotting, whose absence, diminution, or excess may lead to abnormality of the clotting. Twelve factors, commonly designated by Roman numerals, have been described (I-V and VII-XIII; VI is no longer considered to have a clotting function), cofactors such as calcium, phospholipid, vitamin K, and regulators. Regarding regulators of the coagulation cascade, there are five mechanisms that maintain platelet activation and the coagulation cascade regulated: (1) protein C, a major physiological anticoagulant. It is a vitamin K-dependent serine protease enzyme. Protein C is activated in a sequence that starts with protein C and thrombin (protein C is activated by thrombin into activated protein C, APC) binding to a cell surface protein thrombomodulin. Thrombomodulin binds these proteins in such a way that it activates protein C. The activated form, along with protein S and a phospholipid as cofactors, degrades factors Va and VIIIa. Quantitative or qualitative deficiency of either protein C or protein S may lead to thrombophilia, a tendency to develop thrombosis; (2) antithrombin, a serine protease inhibitor (serpin) that degrades the serine proteases: thrombin (factor IIa), factors IXa, Xa, XIa, and XIIa); (3) tissue factor pathway inhibitor (TFPI), it limits the action of tissue factor (TF). It also inhibits excessive TF-mediated activation of factors VII and X); (4) plasmin, it adheres to fibrin in fibrin degradation products that inhibit the excessive formation of fibrin; and (5) prostacyclin, it inhibits the release of granules that would lead to the activation of additional platelets and the coagulation cascade. Anomalies in these mechanisms can lead to an increased tendency toward thrombosis.

This article focuses on antithrombin (AT), a serine protease inhibitor, and is the major plasma inhibitor of thrombin and multiple other coagulation proteases. AT also known as SERPINC1, and is also termed Antithrombin III (AT III). The designations Antithrombin I through to Antithrombin IV originate in early studies carried out in the 1950s by Seegers, Johnson, and Fell [1]. These numbers would imply the following: antithrombin I (AT I) would refer to the adsorption of thrombin on fibrin after thrombin has activated fibrinogen; antithrombin II (AT II) would refer to a cofactor in plasma, which together with heparin interferes with the interaction of thrombin and fibrinogen; antithrombin III (AT III) would refer to a substance in plasma that inactivates thrombin; antithrombin IV (AT IV) would refer to an antithrombin that becomes activated during and shortly after blood coagulation. Only AT III and possibly AT I, are medically significant. While antithrombin III was the original name given to this protein, the correct name now is just antithrombin (AT), with the “III” dropped. The description of AT deficiency in 1965 established the first genetic association between deficiency of a natural anticoagulant and clinical venous thrombosis [2]. This description set into motion the research for other genetic causes for venous thrombosis, with subsequent reports of deficiencies of protein C in 1981 [3], and protein S in 1984 [4],[5]. AT is an important regulator of the coagulation cascade in its function as a serine protease inhibitor, due to inhibition of thrombin and multiple other coagulation proteases. The normal AT concentration in human blood plasma is high at approximately 0.12 mg/ml, which is equivalent to a molar concentration of 2.3 µM [6]. AT has a half-life in blood plasma of around 3 days [7]. Hereditary, AT deficiency is a rare disorder affecting 0.02 to 0.2 percent of the general population [8]–[10]. There are several types of hereditary AT deficiency. Type I deficiency involves any genetic mutation that reduces synthesis or other biochemical mechanisms, resulting in decreased serum levels and activity of AT, often defined by less than 70% of plasma AT antigen (immunoassay) and activity (functional assay) [11],[12]. Type II AT deficiency is a quality defect in the function of AT affecting primarily the thrombin/factor Xa and heparin binding sites. As such, it is identified in patients with normal AT antigen but AT activity <70% of normal [11]. Type II AT deficiency can be divided into three subtypes: genetic mutations affecting the reactive site (RS; IIa), heparin binding site (HBS; IIb), and pleiotropic mutations affecting the s1C–s4B region near the RS (IIc) [11],[13]. Most patients with hereditary AT deficiency are heterozygous, since homozygous variants (except for HBS mutations) are thought to be incompatible with life [11],[13]–[15].

Today the complexity of thrombophilia has grown with appreciation that multiple inherited and acquired risk factors may interact to result in a clinically thrombotic phenotype. This article reviews the pathophysiology and clinical conditions of AT as a serine protease inhibitor with a case history of inherited AT deficiency: Arg393His in AT-Hanoi [16], and discusses the conditions of acquired risk factors such as cancer or genetic disorders via epigenetic modifications in gene-gene (epistasis) and/or gene-environment interactions such as in Lesch-Nyhan disease (LND), in which the β-amyloid precursor protein (APP) that may interact to predispose a patient to thrombosis and cancer as well, and finally, concludes with some future perspectives.

## 2. Case history of AT deficiency of the proband and his family history

**Figure 1.**
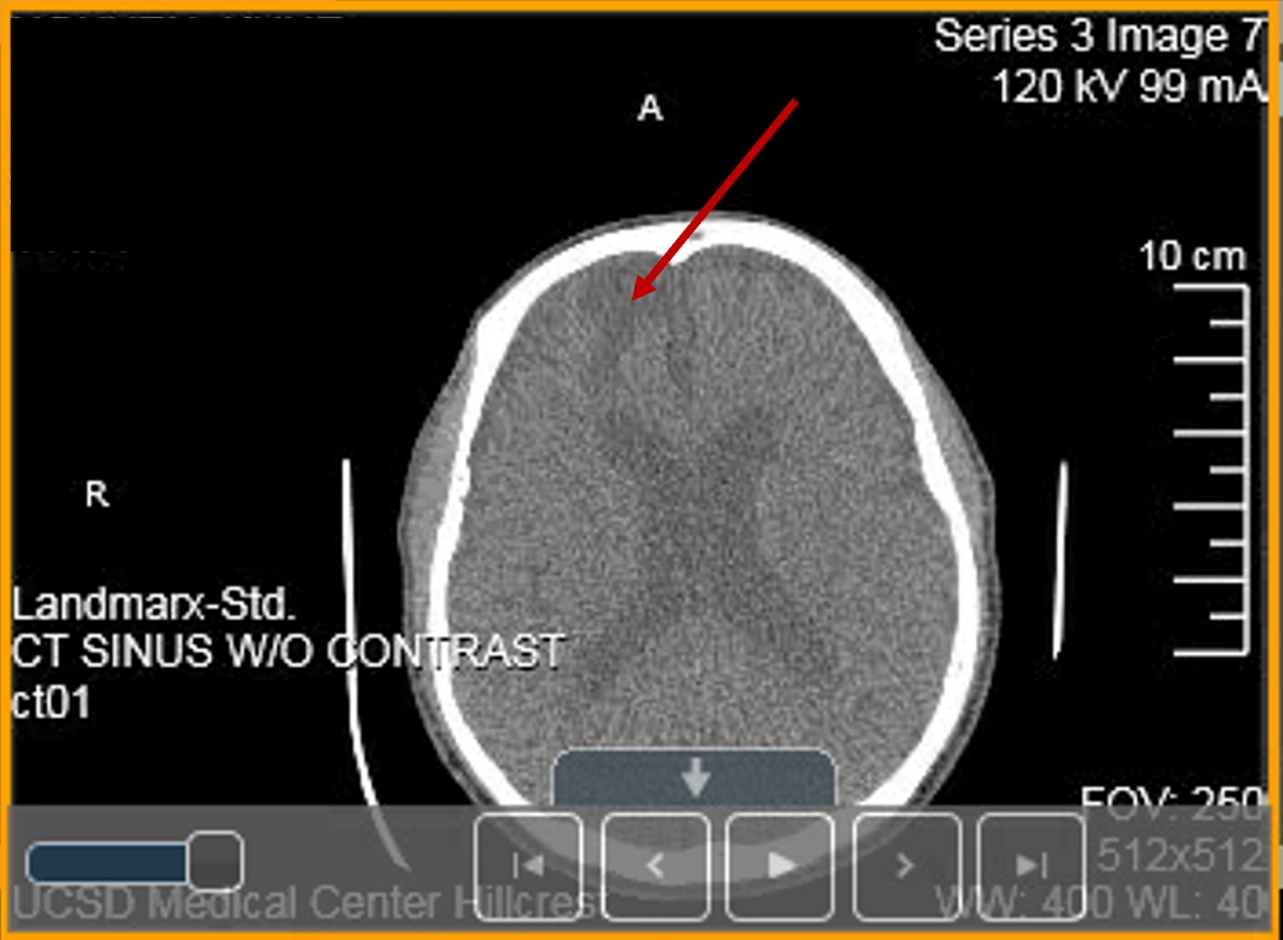
Non-contrast cerebral computed tomography (CT) scans of the paranasal sinuses performed with 0.625 mm axial slices, reformatted in the coronal and sagittal planes. Limited visualized portions of the brain demonstrate encephalomalacia/gliosis involving the anterior right frontal lobe suggesting sequel of prior trauma (dark area at the top-left corner of this image, red arrow).

**Figure 2.**
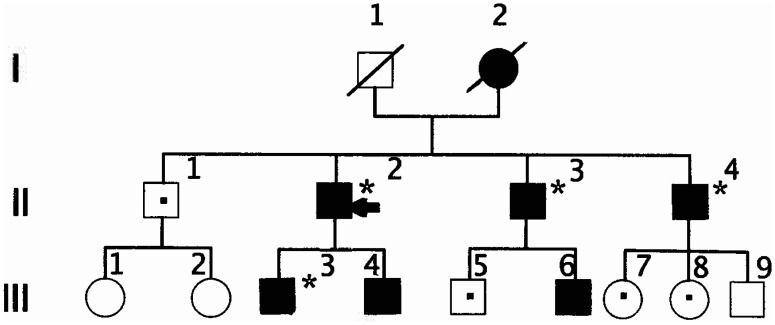
Pedigree of the family. The proband (II-2) is indicated via an arrow. Thrombosis*; Presence of heterozygous Arg393His point mutation, solid symbols; Absence of heterozygous Arg393His point mutation, dotted symbols; Not investigated, open symbols; Deceased, dashed symbols. Male □ Female ○.

The proband (II-2) is a 68-year-old Vietnamese man. He is non-smoking, non-alcoholic, not an obese person, no known allergy to medications, no traumatic brain injury. At age 23, he had a history of idiopathic intracranial hypertension including headache, projecting vomiting with nausea, seizures (epilepsy), visual disturbances, and vertigo. He was admitted to the Regional Besancon Hospital, Besancon, France for evaluation. There were no infectious problems. There were no specific treatments. The cause of this idiopathic intracranial hypertension remained unknown. He remained well after this event. At age 42, he had suffered of swelling and pain in the left leg and deep venous thrombosis (DVT) was confirmed by Doppler ultrasound. He was treated with warfarin (1mg/day). However, just after two weeks of treatment with warfarin (1mg/day), he had suffered of pain in the back and the presence of blood in urine (hematuria) was observed and he was diagnosed with kidney cancer. After surgical removal of the left kidney affected by cancer at the Regional Haguenau Hospital, Haguenau, France, he was treated with warfarin (1mg/day). No specific cancer treatment such as chemotherapy, radiotherapy, etc. was applied after this surgery. He remained well and continued taking warfarin (1mg/day) but he had recurrent DVT at the same site of the left leg as soon as he stopped the treatment with warfarin. He continued then taking warfarin (1mg/day) to prevent blood clots, and since then he has been free of thrombotic event, and free of cancer. When he was 52-year-old, AT deficiency was diagnosed at the Medical Canter of University of California, San Diego, California, U.S.A. and revealed a reduction of functionally active AT to 42% of normal: 50% compared to 118% of the norm (functional AT assay was performed on blood sample of the proband (II-2) using the Sysmex CA-6000, Sysmex Corporation, Kobe, Japan, automated instrument and citrated plasma samples, and Berichrom^®^ Antithrombin-III (A), and Chromogenic method by Date Behring Berrychrome, Newark, DE, U.S.A.). Since then, he was given long-term treatment with warfarin of 3 mg/day to prevent blood clots and maintain an international normalized ration (INR) of 2 to 3 as well as with atorvastatin of 10mg/day to prevent high levels of cholesterol. When he was 63-year-old (July 10, 2015), the factor VIII activity assay was performed on his blood sample at the Medical Canter of University of California, San Diego, California, U.S.A. and found a high value of activity from this factor VIII: 224% compared to the standard range of 55–140%. Recently (August 20, 2019), when he was 67-year-old, from the following up of a history of the right-sided nasal bleeding and red eyes due to complications of the long-term use of warfarin probably, a non-contrast cerebral computed tomography (CT) scan of the paranasal sinuses was performed at the Imaging Services/Radiology, University of California, San Diego Health, California, U.S.A. (CT scanner equipment and radiation dose reduction techniques were employed: CTDIvol: 18.2 mGy, DLP: 282 mGGy-cm and with 0,625 mm axial slices, reformatted in the coronal and sagittal planes). The results obtained showed that the nasal cavity appears clear with no mass lesion or bone destruction, the paranasal sinuses are also clear, no evidence of bony erosion or thickening, but limited visualized portions of the brain demonstrate encephalomalacia/gliosis involving the anterior right frontal lobe suggesting sequela of prior trauma (Figure 1). Then, the cause of the idiopathic intracranial hypertension due to encephalomalacia suddenly occurred at age 23 is now revealed. This finding confirms the presence of a cerebral venous thrombosis (CVT) occurred at age 23 [16]. Currently (68-year-old), he is very well (under long-term treatment with warfarin of 3mg/day to prevent blood clots and maintain an international normalized ration (INR) of 2 to 3 as well as with atorvastatin of 10mg/day to prevent high levels of cholesterol as well as atherosclerosis), free of cancer and thrombotic event. The family pedigree of the proband (II-2) is shown in Figure 2. Other family members related to the mother of the proband (II-2) such as maternal uncles, cousins had had symptoms suggesting AT deficiency such as retinal vein occlusion, cerebral venous thrombosis, and myocardial infarction but were now dead (date not shown).

**Figure 3.**
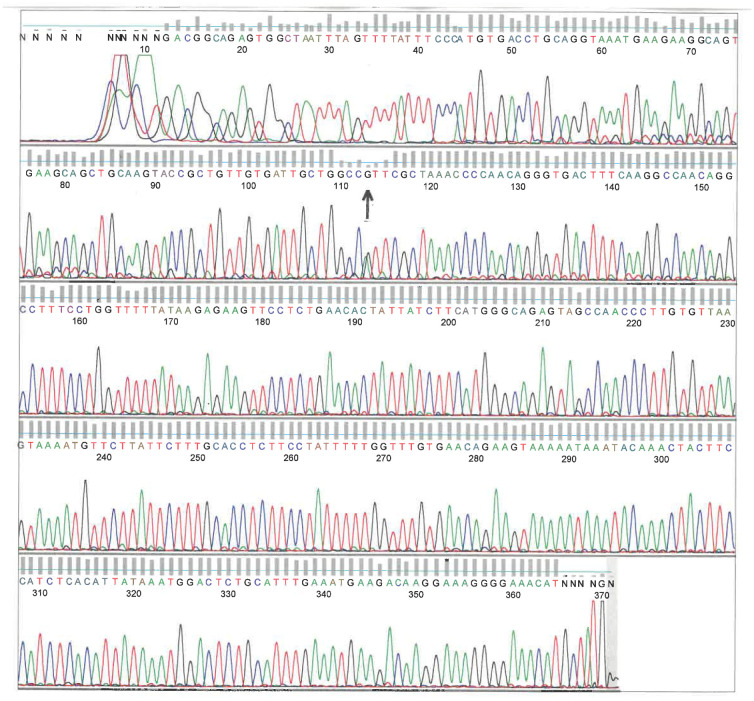
Automated direct DNA sequence analysis of PCR-amplified AT genomic exon/intron fragments of exon 7. The region containing exon 7 (409 bp) from the proband (II-2) was PCR amplified, isolated, purified, and sequenced with the same forward primer as for PCR reaction. Therefore, the sequences presented here are the coding sequence. DNA sequence read from left to right (5′→3′) showed a heterozygous mutation G to A at bp 113 G of the chromatogram (↑). This corresponds to a heterozygous mutation G to A at the nucleotide 13830 of exon 7 (g.13830G > A; c.1274G > A) of the genomic DNA sequence (GenBank X68793) and results in 393Arg (R) → His (H) substitution.

In order to confirm the diagnosis of AT deficiency, the sequencing analysis of the genomic DNA isolated from whole peripheral blood and from buccal cells in mouthwash (Original Mint ScopeR Mouthwash, Procter&Gamble) of the patient (proband II-2) and his different family members was performed. Each of the seven exons and flanking intronic sequences of the human *AT* gene locus (GenBank X68793) were amplified using the polymerase chain reaction (PCR) by means of primers designed to be specific to the exons and franking intronic genomic sequences. Amplification conditions by PCR (denaturation at 94 °C for 1 min, annealing at 60 °C for 2 min, and elongation at 72 °C for 1min, each for 35 cycles) as well as the sequences of the specific primers used (Table 1) were performed as described by Nguyen, K. V. [16]. The results revealed a heterozygous point mutation from both whole peripheral blood and buccal cells in mouthwash (exon 7: g.13830G > A; c.1274G > A; p.393R > H corresponding to Arg393His variant from the proband (II-2) (named as Arg393His in AT-Hanoi for the city in Viet Nam where the proband (II-2) was born) (Figure 3) [16]. This heterozygous Arg393His point mutation was also found from the mother (I-2), the proband brothers (II-3), (II-4), and some of their children (III-3), (III-4), and (III-6) (data not shown).

**Table 1.** Exon-flanking oligonucleotide primer sequences used for the amplification of all seven exons of the human *AT* gene from genomic DNA.

Exon/Length	Primer Name	Nucleotide Sequence (5′ → 3′)	Location (a)
1 (266 bp)	Forward	GAACCTCTGCGAGATTTAGAG	507-527
	Reverse	GTCTTTGACTGTAACTACCAG	752-772
2 (540 bp)	Forward	CTGGAATCCTCTGCTTTACTG	2851-2871
	Reverse	GAGGAATCATTGGACTTGGG	3371-3390
3 (432 bp)	Forward	GGAGTTAACAACTGAGGTGG	5731-5750
	Reverse	CTTCAGCAGCAAAGCAGTGT	6143-6162
4 (370 bp)	Forward	GGCTTCTTAATCAAATGGTGG	6841-6861
	Reverse	GCAGTCCATTTGCCCTCTC	7192-7210
5 (672 bp)	Forward	CCATCATTCTGACACAGCCA	7751-7770
	Reverse	CTAGGATCAGTATCCAGGAG	8403-8422
6 (308 bp)	Forward	GTGAGAGTATGATTAGGTGAAG	10189-10210
	Reverse	GCATGCCTTAACACTGGAAAC	10476-10496
7 (409 bp)	Forward	GGAATTGCTGTGTCTGTGGA	13691-13710
	Reverse	CCATGTGCCCCAATAGCATG	14080-14099

(a) Exon-flanking oligonucleotide primer sequences numbering is based on GenBank X68793.

In addition, the sequencing analysis after PCR amplification obtained for ten exons and flanking intronic sequences of the tumor suppressor protein p53 (*TP53*) (GenBank X54156) and the human homologue of the murine double minute 2 protein (*HDM2*) (GenBank NC_000012.11) genes from genomic DNA isolated from whole peripheral blood of the proband (II-2) revealed no mutations (data not shown) [16]. The sequences of primers used for PCR are available upon request.

In sum, as shown in Figure 2, the proband's mother (I-2) died at age of 97-year-old and carrier of AT deficiency, was asymptomatic. The proband (II-2) had had CVT (encephalomalacia) at age 23 and had suffered DVT of the leg and developed kidney cancer at age 42. Both of two his children: (III-3) (37-year-old) and (III-4) (35-year-old) showed the presence of Arg393His point mutation, in which the one (III-3) had developed DVT of the leg at age 35 while the other (III-4) is actually no symptoms of AT deficiency. The proband's brother (II-3) (66-year-old) showed the presence of heterozygous Arg393His point mutation, had suffered DVT of the leg and mesenteric venous thrombosis at age 50. His AT plasma concentration is not available. One of the two his children (III-6) (34-year-old) showed the presence of heterozygous Arg393His point mutation, is actually no symptoms of AT deficiency. The proband's brother (II-4) (64-year-old) showed the presence of heterozygous Arg393His point mutation, had suffered DVT of the leg at age 60. His AT plasma concentration is not available.

## 3. Discussion

### 3.1. AT structure, function, AT deficiency, and AT management

#### 3.1.1. AT structure and function overview

**Figure 4.**
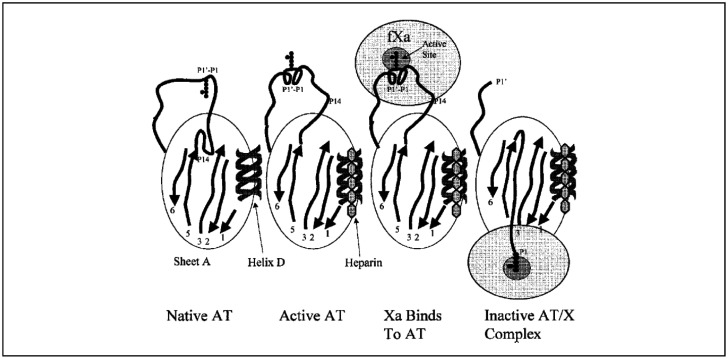
This drawing shows the mechanism of antithrombin (AT) inhibition of factor Xa. Antithrombin, like other serpins, is able to inactivate factor Xa by forming a covalent 1:1 complex with the serine protease, a process termed suicide substrate inhibition. The tertiary structure of antithrombin includes a 5-stranded central (sheet A; strands 1,2,3,5, and 6), together with a heparin-binding D-helix and a mobile reactive site loop (P1-P17′ shown as a black loop at the top of the molecule). The reactive site loop includes a scissile P1-P1′ (Arg393-Ser394) bond that resembles the substrate for thrombin and other serine proteases. In its native state, antithrombin inactivates factor Xa inefficiently, due to conformational inaccessibility of the P1-P1′ bond (shown as pointing downward on the diagram). Inhibition is accelerated approximately 1000-fold by the binding of heparin to arginine residues in the D-helix of antithrombin, with a resultant conformational change of the reactive site loop and exposure of the P1-P1′ reactive center (shown as pointing upward). Once the factor Xa cleaves the bond, the protease is covalently linked to the P1 residue, and the reactive loop peptide becomes mobile. The reactive loop peptide then hinges and incorporates into the central β-sheet, becoming a six strand. This induces a hingelike translocation of factor Xa to the distal end of the antithrombin molecule and its inactivation due to geometric distortion of the active site.

AT is a single-chain plasma glycoprotein, which is a heparin cofactor and member of the serine protease inhibitor (collectively known as serpin) gene family. AT is a natural anticoagulant that inhibits thrombin (factor IIa), factor Xa (briefly, thrombin (factor IIa), is a serine protease, an enzyme EC 3.4.21.5 that, in humans, is encoded by the *F2* gene. Prothrombin, coagulation factor II, is proteolytically cleaved to form thrombin in the clotting process. Thrombin in turn acts as a serine protease that convers soluble fibrinogen into insoluble strands of fibrin, as well as catalyzing many other coagulation-related reactions. Factor Xa is the activated form of the coagulation factor X. Factor X, also known by the eponym Stuart-Prower factor, is an enzyme EC 3.4.21.6, of the coagulation cascade. It is a serine endopeptidase, protease group S1 peptidase family. Factor X is synthesized in the liver and requires vitamin K for its synthesis. Factor X is activated, by hydrolysis, into factor Xa by both factor IX (with its cofactor: factor VIII in a complex known as *intrinsic Tenase*) and factor VII (with its cofactor: tissue factor, a complex known as *extrinsic Tenase*). Factor Xa plays a critical role in the coagulation cascade by catalyzing the proteolytic conversion of protrombin to active thrombin in conjunction with other cofactors), and other serine proteases in the coagulation cascade [17]. As a serine protease inhibitor, its activity is accelerated more than 1000-fold by heparin binding [8]. In the absence of AT, heparin has little effect on anticoagulation [8],[18]. Several different antithrombin activities in plasma were reported during the first half of the 20^th^ century [19],[20], leading to the classification of antithrombins I through IV [21]. It was subsequently shown that these various antithrombin activities were actually the function of one molecule, antithrombin III (AT III), whose name was shortened to simply antithrombin (AT) at the 1993 meeting of the International Society on Thrombosis and Haemostasis [22],[23]. AT gene is located on human chromosome 1 (q23-25) [24]. This AT gene spans 13480 bp of the DNA from the transcription start site to the poly A signal, and has 7 exons [25]–[27]. The mature AT molecule has a molecular weight of 58200 Daltons with 432 amino acids [28],[29]. The liver is the primary source of AT synthesis and posttranslational glycosylation [30]. AT, like other serpins, is able to inactive thrombin by forming a covalent 1:1 complex with the serine protease, a process termed suicide substrate inhibition [31]. Reaction between AT and serine proteinases is catalyzed by heparin which induces a conformational change in the inhibitor and also provides a template for “approximation” of inhibitor and enzyme during its accelerated inhibition. Appreciable evidence indicates that the amino-terminal region of AT is involved in its interaction with heparin. Furthermore, it has been demonstrated that thrombin attacks a specific reactive bond of AT near its COOH terminus during AT-thrombin complex formation. This bond has been identified as Arg393-Serin394 and is the active site of AT [32]. Both the P1 (Arg393) and P′1 (Ser394) positions are critical for serpin activity [33]. The minimum heparin species necessary for inducing the conformational change in AT has been determined to be a specific pentasaccharide sequence. While the pentasaccharide is sufficient to accelerate the inhibition of factor Xa, the inhibition of thrombin requires a bridging contribution from heparin and the formation of a trimolecular complex between AT, thrombin, and the heparin species [33]. A drawing represents the mechanism of AT inhibition of factor Xa is shown in Figure 4 [33].

#### 3.1.2. AT deficiency

The existence of a deficiency state involving AT was recognized first by Egeberg who described a Norwegian family with recurrent episodes of venous thromboembolism (VTE), which includes both DVT and pulmonary embolism (PE) [2]. Since then, families with hereditary AT deficiency have been found in many countries, and the link between AT deficiency and thrombosis has now been clearly established [34],[35]. AT deficiency generally be recognized when a patient suffers recurrent VTE. AT deficiency is typically considered to have an autosomal dominant mode of inheritance, but some genetic heterogeneity does exist. It presents as a heterozygous state almost exclusively, the homozygous state being extremely rare and usually lethal, presenting with neonatal thrombosis [36]. Individuals with AT deficiency have been shown to have an increased risk for thrombosis, ranging from 5-fold to a 50-fold increase [33]. Thrombosis in individuals with AT deficiency is rare in the first decade, but the initial thrombotic event usually occurs before the age of 30 [33],[36]. Congenital variants resulting in clinical morbidity have been described such as AT-Toyama (R47C) [37], AT-Basel (P41L) [38], and AT-Rouen (R47H) [39] are defective in heparin binding but have near normal “progressive” activity in the absence of heparin. AT-Denver (S394L) [40], AT-Northwick Park (R393C) [41],[42], AT-Glasgow (R393H) [43],[44], AT-Sheffield (R393H) [45], AT-Kumamoto (R393H) [46], and AT-Hanoi (R393H) [16] are defective in serpin activity but bind heparin normally. The present Arg393His point mutation in AT-Hanoi is characterized by a functionally active AT so reduced (42% of normal) resulting in multiple severe thrombotic events such as an idiopathic intracranial hypertension suddenly occurred at age 23 due to a CVT (encephalomalacia/gliosis revealed at age 67 via a non-contrast CT scan) [16],[47], recurrent DVT and the development of kidney cancer (occurred at age 42) (see case history report).

Arginine and histidine, both are polar, hydrophilic and basic molecules. The side chain of arginine consists of a 3-carbon aliphatic straight chain ending in a guanidine group while it is an imidazole for histidine. On charge considerations alone, arginine's side chain is amphipathic (i.e. having both hydrophilic and hydrophobic parts), because at physiologic pH, it contains a positively charged guanidinium group, in which is highly polar at the end of a hydrophobic aliphatic hydrocarbon chain. Because globular proteins have hydrophobic interiors and hydrophilic surfaces [48], arginine is typically found on the outside of the protein, where the hydrophilic head group can interact with the polar environment, for example taking part in hydrogen bonding and salt-bridges that can be important for protein stability. For this reason, it is frequently found at the interface between two proteins. The aliphatic part of the side chain sometimes remains below the surface of the protein or to be buried in the protein core. Arginines are then quite frequent in protein active or binding sites. The positive charge means that they can interact with negatively charged non-protein atoms (e.g. anions or carboxylate groups). Arginine contains a complex guanidinium group on its side chain that has a geometry and charge distributions that is ideal for binding negatively-charged groups on phosphates (it is able to form multiple hydrogen bonds). Concerning histidine, its side chain is partially protonated at physiological pH (imidazole's side chain of histidine has a pKa of approximately 6.5 at physiological pH, which means that only about 10% of molecules will be protonated. The precise pKa depends on local environment. It is therefore false to presume that histidine is always protonated at typical pHs). The fact that histidine has a pKa near to that of physiologic pH, meaning that it is relatively easy to move protons on and off the side chain i.e. changing the side chain from neutral to positive charge. This flexibility has two effects. The first is ambiguity about whether it prefers to be buried in the protein core or exposed to solvent. The second is that it is an ideal residue for protein funtional centres. Histidines are therefore the most common amino acids in protein active or binding sites. They are very common in metal binding sites (e.g. zinc), often acting together with cysteines or other amino acids. In this context, it is common to see histidine replaced by cysteine [48]. Taking into consideration these remarks, the substitution of arginine by histidine in Arg393His point mutation would affect severely the inhibition activity of AT vis a vis thrombin. Indeed, at physiologic pH, there are only about 10% of histidine will be protonated to be positive charge [64] needed for binding to thrombin at the active site Arg393-Serin394 of AT. This would affect consequently the inhibition activity of AT vis a vis thrombin (judged by functional assay) although the AT plasma concentration is normal (judged by immunoassay), and so that in the present Arg393His point mutation of AT-Hanoi, there is a dramatically reduction of functionally active AT to 42% of normal resulting in multiple severe thrombotic events [16]. In addition, high plasma level of factor VIII found from the proband (II-2): 224% compared to the standard range of 55–140% (see case history report) suggests an increase in the risk of thrombotic event [49],[50]. Arg393His point mutation affecting the active site of AT (Arg393-Ser394) [32] is considered as the one with high incidence of thrombosis [16],[43]–[46].

One of the extremely serious medical conditions that cause damage to the brain of a person, especially to the proband (II-2), is encephalomalacia, also known as cerebral softening [51]. It can occur in anyone regardless of race, age, or gender, including infants and the embryo during development. This condition is fatal for infants. Encephalomalacia can spread to areas of the brain that are adjacent or appear in a particular part of the brain. In this condition brain tissue mainly parenchyma become soften and liquid. Encephalomalacia is the outcome of parenchyma tissue necrosis causes softening and liquefactive of the part of brain. This effect may happen at one part of the brain and then extend into adjacent tissues. Encephalomalacia is a localized softening of the substance of the brain, due to bleeding or inflammation. However, in rare cases, it can spread to other parts of the organ. The softening can affect the brain in numerous ways, especially when it leads to scarring that often causes further brain issues. While it is uncommon, softening of the entire brain does occur as well as complete shutdown of the parts of the brain affected by encephalomalacia. In other words, this condition is deadly serious and should be treated as such. There are two different types of encephalomalacia: polioencephalomalacia and leukoencephalomalacia. Polioencephalomalacia affects the gray matter of the brain. Gray matter is an important part of the central nervous system (CNS) (about 40% of the brain is gray matter), affecting things like memory, emotions, muscle control, speech, and sensory perception. Leukoencephalomalacia affects the white matter, which the brain uses for transmitting signals from one end of the cerebrum to another. There are also three different categories of softening distinguished by their color and representing different stages of the disease progress: red, yellow, and white softening [52],[53]. Red softening, as its name suggests, certain regions of cerebral softening result in a red color. This is due to the softening occurs in an area of the brain where blood flow is restored but was previously restricted by a fat globule, blood clot, foreign material, or gas bubble in the bloodstream. This is termed a “red infarct” or also known as red softening [52]. Yellow softening, as its name implies, the affected softened areas of the brain have a yellow appearance. This yellow appearance is due to a buildup of atherosclerotic plaque in the interior brain arteries coupled with yellow lymph around the choroid plexus, which occurs in specific instances of brain trauma [53]. White softening occurs in the area of the brain that does not have much in the way of blood flow. There are known as “pale” or “anemic infarcts” and are areas that contain dead neuronal tissue, which result in a softening of the cerebrum [52]. There are many different encephalomalacia causes. The most common causes of encephalomalacia include cerebral infarction and ischemia, infection, and traumatic brain injury. Cerebral infarction is a technical term for what we generally refer to as a stroke. It is an interruption of blood flow to the brain by an obstruction (blood clot, for example). Cerebral ischemia is similar as it is a reduced flow of blood to the brain due to obstruction; this usually results in mini-strokes. A number of infections that either spread to the brain or directly affect the organ can cause damage resulting in the softening of the brain's tissues [54],[55]. One of the most common causes of brain softening is traumatic brain injury. This can range from a car accident to a bad fall to being shot in the head. In the case of the proband (II-2): a non-contrast CT scan of the paranasal sinuses demonstrated encephalomalacia/gliosis involving the anterior right frontal lobe suggesting sequela of prior trauma. Gliosis is a nonspecific reactive change of glial cells in response to damage to the CNS. The process of gliosis involves a series of cellular and molecular events that occur over several days [56]. The final component of gliosis is astrogliosis, the proliferation of surrounding astrocytes, which are the main constituents of the glial scar formation (Figure 1). Then, the cause of the idiopathic intracranial hypertension due to encephalomalacia suddenly occurred at age 23 is now revealed. This finding confirms the presence of CVT occurred at age 23 related to the Arg393His point mutation of AT identified later [16] (see case history report). Sadly, there is no direct treatment or cure for encephalomalacia. Once the brain is damaged, it is damaged permanently!

#### 3.1.3. AT deficiency management

Inherited as an autosomal dominant trait, congenital AT deficiency typically reduces functional AT levels to 40–60% (often defined as less than 70%) of normal. As a result, individuals with hereditary AT deficiency have a ≥50% lifetime risk of VTE, which includes both DVT and PE [2]. Specifically, AT deficiency is associated with a three-to seven-fold higher risk of VTE compared with other thrombophilias. The management of VTE is based upon balancing the treatment benefits against the risk of bleeding from the treatment. Thus, maintaining adequate levels of AT during high-risk periods is an important treatment goal. Long-term anticoagulant thromboprophylaxis is not recommended in asymptomatic patients with AT deficiency because of the increased risk of hemorrhage. However, treatment guidelines recommend short-term thromboprophylaxis in high-risk clinical settings, including surgery, trauma, and management of pregnancy, labor, and delivery. The role of treatment for patients with hereditary AT deficiency is an initial increase in AT activity to ≥120% of normal levels followed by maintenance of AT activity at ≥80% of normal levels [15]. For such a purpose, different treatments are used:

- Heparin and low-molecular-weight heparin (LMWH) are the most commonly administered anticoagulants in high-risk clinical settings [15],[33]. Heparin alone has no direct anticoagulant effect but potentiates the activity of AT by enhancing AT-mediated inhibition of coagulant enzymes more than 1,000-fold as the result of a conformational change in AT. Commercially available heparins are prepared from porcine intestinal mucosa or bovine lungs, largely because they are a good source of heparin-rich mast cells. In general, most commercial heparin preparation are heterogeneous and have a molecular weight between 7000 and 25000 Daltons, make up polydisperse pharmaceutical-grade heparin with the pentasaccharide sequence comprising only approximately 30% of the mass. These larger heparin species are able to accelerate the inhibition of both factor Xa and thrombin, resulting in a narrow therapeutic window and risk of bleeding with excess heparin [33]. In addition, patients with AT deficiency would be also expected to exhibit heparin resistance [15]. LMWHs, in contrast, consist of only short chains of polysaccharide. LMWH species such as reviparin, enoxaparin, dalteparin, etc. prepared by chemical or enzymatic degradation, have a molecular weight of approximately 5000 Daltons and primarily inhibit factor Xa [33]. Because LMWH can be given subcutaneously and does not require the partial thromboplastin time (PTT) or activated partial thromboplastin time (aPTT or APTT) monitoring (PTT or aPTT or APTT is a blood test that characterizes coagulation of the blood), it permits outpatient treatment of conditions such as DVT or PE that previously mandated inpatient hospitalization for unfractionated heparin (UFH) administration. LMWHs inhibit the coagulation process through binding to AT via a pentasaccharide sequence. This binding leads to a conformational change of AT which accelerates its inhibition of activated factor X (factor Xa). Once dissociated, the LMWH is free to bind to another AT molecule and subsequently inhibit more activated factor X. Unlike AT activated by heparin, AT activated by LMWH cannot inhibit thrombin, but can only inhibit clotting factor Xa [33]. The effects of LMWHs cannot be then acceptably measured using PTT or activated clotting time (ACT) tests. Rather, LMWH therapy is monitored by the anti-factor Xa assay, measuring anti-factor Xa activity rather than a clotting time. The methodology of an anti-factor Xa assay is that patient plasma is added to a known amount of excess recombinant factor X and excess AT. If heparin or LMWH is present in the patient plasma, it will bind to AT and form a complex with factor X, inhibiting it from becoming factor Xa. The amount of residual factor Xa is detected by adding a chromogenic substarte that mimics the natural substrate of factor Xa, making residual factor Xa cleave it, releasing a colored compound that can be detected by a spectrophotometer. AT deficiencies in the patient do not affect the assay, because excess amounts of AT is provided in the reaction [57].

- Fresh frozen plasma (FFP) is an additional treatment option for individuals with hereditary AT deficiency; however, clinical evidence demonstrating its efficacy is lacking. In addition, safety issues are associated with the administration of human blood products. Transmission of viral infections, such as human immunodeficiency virus (HIV), West Nile virus (WNV), hepatitis B, and hepatitis C is a small but not insignificant risk associated with transfusion of homologous blood products such as FFP. In addition to risks associated with viral transmission, there remains a risk of clerical errors associated with collection, storage, and administration of blood products. Furthermore, prions that cause variant Creutzfeldt-Jacob disease are carried in plasma, but the risk of transfusion-related transmission is uncertain [15].

- Moreover, AT concentrate is also an additional treatment option for individuals with hereditary AT deficiency [15],[58]. Two types of AT concentrates are commercially available. AT concentrate derived from the pooled human plasma of healthy donors is indicated in the treatment and prevention of VTE in patients with hereditary AT deficiency whose AT activity is less than 70%. Recombinant human AT concentrate, derived from the milk of genetically engineered goats, is approved in the United States for the prevention of perioperative and peripartum thromboembolic events in hereditary AT deficient patients. The most common (>5%) adverse reactions from AT concentrate infusion include hemorrhage (intra-abdominal, hemarthrosis, and postprocedural), and infusion site reaction. The risk of transmission of infectious agents with human plasma-derived AT concentrate is low, and serious adverse events have not been reported in the literature [15],[58].

- Otherwise, there are also other drugs named as vitamin K antagonists (VKAs). They are a group of substances that reduce blood clotting by reducing the action of vitamin K. The term “vitamin K antagonist” is technically a misnomer, as the drugs do not directly antagonize the action of vitamin K in the pharmacological sense, but rather the recycling of vitamin K. They are used as anticoagulant medications in the prevention of thrombosis, and in pest control, as rodenticides. These drugs deplete the active form of the vitamin by inhibiting the enzyme vitamin K epoxide reductase and thus the recycling of the inactive vitamin K epoxide back to the active reduced form of vitamin K. The drugs are structurally similar to vitamin K and act as competitive inhibitors of the enzyme. Coumarins (more accurately 4-hydroxycoumarins) are the most commonly used VKAs. In medicine, the most commonly used VKA is warfarin [59]. The name warfarin stems from its discovery at the University of Wisconsin, incorporating the acronym for the organization that funded the key research, WARF for the Wisconsin Alumni Research Foundation and the ending –arin, indicating its link with coumarin. In some countries, other coumarins are used instead of warfarin, such as acenocoumarol and phenprocoumon. These have a shorter (acenocoumarol) or longer (phenprocoumon) half-life, and are not completely interchangeable with warfarin. Warfarin was initially used as a rodenticide, but made the transition to pharmaceutical. It is sold under brand name Coumadin among others, and is a medication that is used as an anticoagulant. It is referred to as “blood thinner”, this is a misnomer since it does not affect the viscovity of blood. Warfarin decreases blood clotting by blocking an enzyme called vitamin K epoxide reductase that reactivates vitamin K_1_ [56]. Without sufficient active vitamin K_1_, clotting factors II, VII, IX, and X have decreased clotting ability [60]. The anticlotting protein C and protein S are also inhibited, but to a lesser degree [60]. Warfarin treatment can help prevent formation of future blood clots and help reduce the risk of embolism (migration of a thrombus to a spot where it blocks blood supply to a vital organ [61]. It is commonly used to treat blood clots such as DVT and PE, and to prevent stroke in people who have atrial fibrillation, valvular heart disease or artificial heart valves [62]. It is generally taken by mouth, but may also be used by injection into a vein [62]. The common side effect of warfarin is bleeding [62]. The risk of severe bleeding is small but definite (a typical yearly rate of 1–3% has been reported). All types of bleeding occur more commonly, but the most severe ones are those involving the brain (intracerebral hemorrhage/hemorrhagic stroke) and spinal cord. To optimize the therapeutic effect without risking dangerous side effects such as bleeding, it is recommended that the effects of warfarin typically monitored by checking prothrombin time (PT) or international normalized ratio (INR) every one to four weeks [62]. The target INR level varies from case to case depending on the clinical indicators, but tends to be 2–3 in most conditions. Risk of bleeding is increased if the INR is out of range (due to accidental or deliberate overdose or due to interactions). This risk of bleeding increases greatly once the INR exceeds 4.5. Less common side effects of warfarin may include areas of tissue damage and purple toes syndrome [62]. Use of warfarin is not recommended during pregnancy [62]. The metabolism of warfarin varies greatly between patients. Many other medications and dietary factors can interact with warfarin, either increasing or decreasing its effectiveness [60],[62]. Many commonly used antibiotics, such as metronidazole or the macrolides will increase the effect of warfarin by reducing the metabolism of warfarin in the body. Food that contains large quantities of vitamin K_1_ will reduce the warfarin effect (leafy green vegetables tend to contain higher amounts of vitamin K_1_; foods low in vitamin K_1_ include roots, bulbs, tubers, cereals, grains, and other milled products, most fruits and fruit juices). In addition, excessive use of alcohol is also known to affect the metabolism of warfarin and can elevate the INR and thus increase the risk of bleeding [63]. When taken with nonsteroidal anti-inflammatory drugs (NSAIDs), warfarin increases the risk for gastrointestinal bleeding. This increased risk is due to the anti-platelet effect of NSAIDs as well the possible damage to the gastrointestinal mucosa [64]. Dosing of warfarin is then complicated. The maintenance dose of warfarin can fluctuate significantly depending on the amount of vitamin K_1_ in the diet. Keeping vitamin K_1_ intake at a stable level can prevent these fluctuations.

It was reported that the incidence of VTE in Asian populations is lower than in Western countries [65]. In fact, although the clinical assessment, diagnostic testing, and therapeutic considerations for VTE are, in general, the same in Asia populations as they are in Western populations, the overall burden of VTE in Asia has been considerably underestimated. In both Asian and Western populations, compression ultrasound (CUS) and multidetector computed tomographic angiography (CTA) have become the methods of choice for effectively imaging the vasculature with high sensitivity and specificity in patients with suspected DVT and PE, respectively [65]. Factors that may explain the lower prevalence of VTE in Asia populations relative to Western populations include the low awareness toward thrombotic diseases, under-diagnoses, low autopsy rates-mainly because of cultural and religious practices, limited availability of epidemiological data in Asia, ethnic differences in genetic predisposition to VTE (such as obesity increases the propensity to thrombosis, the leading cause of death in the Western World [66], and mutations: factor V Leiden [67] and prothrombin G20210A polymorphisms [68] are exclusive to Caucasians while the prevalence of protein C [3], protein S [4],[5], and antithrombin [2] deficiencies in Asian populations are higher than those found in Caucasians), and possibly less symptomatic VTE in Asian patients (the thrombi tend not to advance to symptomatic thrombosis in Asian patients) [65]. Regarding the AT management, the Asian Venous Thrombosis Forum recommended the use of mechanical prophylaxis such as graduated compression stockings, pneumatic compression devices, foot pumps, etc. for patients with increased risk of bleeding [69], and mechanical prophylaxis in combination with pharmacological prophylaxis including LMWH, fondaparinux, dabigatran, apixaban, rivaroxaban, low dose of UFH, VKA, aspirin, or non-vitamin K antagonist oral anticoagualnts (NOACs) (NOACs include the direct thrombin inhibitor dabigatran and the direct factor Xa inhibitors rivaroxaban, apixaban, and edoxaban. NOACs may simplify patient management in Asia primarily due to no regular coagulation-monitoring requirement because of their predictable pharmacokinetic (PK) and pharmacodynamics (PD) properties. They can be taken orally in fixed doses once or twice daily, and they have minimal food and drug-drug interactions and demonstrating no interactions with NSAIDs) for patients with high risk of VTE [70]. NOACs have been approved for the treatment of VTE in many countries in Asia; however, only a few countries provide reimbursements to patients. In any way, the lack of clinical trials assessing the efficacy and safety of NOACs for the treatment and prevention of VTE specifically in Asian populations make it difficult to change the standard of care in Asian countries [65].

### 3.2. Acquired risk factors for thrombosis

#### 3.2.1. Cancer and thrombosis

VTE is a highly prevalent and potentially fatal disease. It is the third most common cause of cardiovascular death, following acute corona artery disease and stroke, and is responsible for more than 3 million deaths per year worldwide [71]. Several risk factors have been associated with VTE such as obesity [66],[72], inflammation [73], severe acute respiratory syndrome coronavirus 2 (SARS-CoV-2: COVID-19) [74],[75], hormone use [76], and immobility among others [77], but none as relevant as cancer [78]. Cancer is a major cause of death in VTE patients and *vice versa* [79]. The history of the relationship between cancer and thrombosis dates back to 1823 with a paper that appears to be the fist report of an association between cancer and thrombosis published by the French physician Jean-Baptiste Bouillaud [80]. In 1865, another French physician Armand Trousseau reported an association between gastric cancer and VTE [81]. Close reading of his papers reveal that all his patients described with VTE already had extensive evidence of cancer at the time of the diagnosis of thrombosis [82],[83]. According to the Anglo-Saxon literature, the honor of the first description of a patient with DVT and the manifestation of a gastric cancer several months later was from the report of Illyd James and Matheson in 1935 [84]. Interestingly, the first proper cohort study in patients with VTE to assess the incidence of occult cancer was only published in 1982 [85]. All together, these reports considered the beginning of attention that malignant disease and hemostasis interact together, and since then researchers have been increasingly recognized the “two way association” idea of the relationship between cancer and thrombosis in which VTE can both a presenting sign and complication of cancer. Cancer is responsible for 18% of all case of incident VTE. Across all patients with cancer, the risk for VTE is elevated 7-fold; in certain malignancies, the risk for VTE may be increased up to 28-fold [79]. Also, on the basis of statistical results from cohort studies and clinical trials provided considerable evidence for a two way clinical association between VTE and cancer: cancer therapy itself has been shown to increase the risk for VTE whether it be chemotherapy, antiangiogenic therapy, or hormonal therapy, particularly during the first few months after diagnosis and in the presence of distant metastases; and from a series of patients hospitalized for VTE, it has been reported that thrombotic episodes may also precede the diagnosis of cancer by months or years thus suggesting that thrombosis may be the first clinical manifestation of an occult malignancy, in some patients [79],[86]–[88]. In patients with symptomatic VTE, the prevalence of concomitant cancer (i.e. cancer not known before the diagnosis of VTE and discovered by routine investigation) at the time of VTE diagnosis varies considerably between the studies due to differences in, for instance, threshold of suspicion, screening method, and characteristic of the patients like age, and found, in the larger studies, between 4% and 12% [89]. Furthermore, the risk of concomitant cancer is 3-to 4-fold increased in patients with idiopathic VTE compared with secondary VTE [89]. The risk of occult cancer (i.e. cancer that becomes clinically apparent during follow-up) is also increased in patients with VTE. The prevalence of occult cancer in patients with idiopathic VTE is 4–10% while the prevalence of occult cancer in patients with secondary VTE is comparable with the prevalence of cancer in the general population [89]. The risk of developing overt cancer after VTE also depends on the type of cancer. In the large Danish registry, Sorensen and colleagues showed that around 15% of the patients with VTE and cancer within 1 year had lung cancer, followed by prostate (11.4%), pancreas (7.9%), colon (7%), and breast (4.3%) cancer [90]. In addition, there is also thrombogenic mechanism that is mediated via antiphospholipid antibodies (aPL) [91]. Higher prevalence of aPL was observed in patients with solid tumors compared to controls. aPL presence may be a risk factor for malignancies (particularly hematological). One of the most severe complications of aPL presence is the Catastrophic Antiphospholipid Syndrome (CAPS). Among CAPS associated cancers lymphomas and leukemias are the most representative group [91]. In fact, the association between CAPS and these malignancies underline the prevalent pathogenetic mechanism from small vessels thrombosis [91]. In neoplastic patients, aPL presence can increase thromboembolic risk and in healthy carriers, can increase the possibility of developing a malignancy [91]. Considering the high incidence of cancer in the first months after VTE, screening for an underlying malignancy may be clinically relevant.

Several recent studies have reported the incidence of VTE among cancer patients in Asia. The most common cancer associated VTE in Thailand were gynecologic cancers, followed by gastrointestinal and hepatobiliary cancers, lung cancer, and lymphoma [92]. A Taiwanese population-based study investigating the relationship between unprovoked VTE and cancer risk showed that the risk of cancer was significantly higher in the unprovoked VTE patients (hazard ratio 2.3). The risk was increased in the 6 months after VTE. Therefore VTE can be a presenting symptom of occult cancer [92].

It has been postulated that clot formation at the tumor periphery may: (1) facilitate attachment of metastasis tumor cells to endothelial cells (tumor cells which fail to adhere do not survive); (2) provide nutrients and/or growth stimulants; (3) serve as structural lattice upon which tumor cells can proliferate; or (4) protect the tumor cells from host defense mechanisms [93]. Patients with cancer are at higher risk of thromboembolic complications than healthy people for many reasons. First, there is a complex relationship between cancer and host cells that troubles the balance between coagulation and fibrinolysis. Secondly, tumor needs of new blood vessels to grow, but proangiogenic factors as vascular endothelial growth factor can also promote a thrombophilic state by causing the secretion of procoagulant substances from endothelial cells. Then, tumor cells can active blood coagulation through multiple mechanisms, including production of procoagulant, fibrinolytic, and proaggregating activities, release of proinflammatory and proangiogenic cytokines, and interacting directly with host vascular and blood cells (e.g. endothelial cells, leukocytes, and platelets) through adhesion molecules. Increasing evidence suggests that elements of the haemostatic system also have a direct role in eliciting or enhancing angiogenesis, cell survival, and metastasis [79].

The VTE is now considered to be a chronic disease, in that the risk for recurrent persists for many years after the initial event. For many decades, experimental and clinical studies have evaluated the effects of anticoagulants on tumor growth with different outcomes. The treatment of VTE in cancer patients aims at reducing mortality and morbidity, and improving quality of life. Until the mild-2000s, the standard treatment for acute VTE consisted of initial therapy with LMWH or UFH followed by long-term therapy with VKAs [94],[95]. Both UFH and the LMWH are recommended for primary prophylaxis following cancer surgery. Studies show that LMWHs are at least as effective as UFH in this setting, but associated bleeding tendency is lower than UFH [94],[95]. LMWH is preferred as an effective and safe for treatment of VTE. It has largely replaced UFH and VKAs because LMWH does not need hospitalization and laboratory monitoring like UFH. Also, LMWH is associated with a lower risk of heparin-induced thrombocytopenia (HIT) (HIT is the development of thrombocytopenia i.e. a low platelet count, due to the administration of various form of heparin. HIT predisposes to thrombosis because platelets release microparticles that activate thrombin, thereby leading to thrombosis. HIT is caused by the formation of abnormal antibodies that activate platelets. The treatment of HIT requires stopping heparin treatment, and both protection from thrombosis and choice of an agent that will not reduce the platelet count and further. Several alternatives are available for this purpose; mainly used are danaparoid, fondaparinux, argatroban, and bivalirudin) and simple dosing (once-daily, weight-based subcutaneous injection) [95]. VKAs have been the mainstay agents for long-term management and secondary prophylaxis of acute VTE in patients without cancer. However, it was reported that warfarin is associated with a high bleeding rate in patients with VTE and cancer despite maintenance of the INR within the therapeutic range. In addition to lower efficacy, VKAs also need laboratory monitoring of their anticoagulant activity; and their absorption affected by food interactions [94],[95]. LMWHs are recommended for use in secondary/long-term prophylaxis where, compared with warfarin, they display increased efficacy with a good safety profile and reliability, and are associated with increased quality of life [94],[95]. Furthermore, LMWHs have been associated with potential antineoplastic effects that may contribute to improved survival times in cancer patients [95]. However, abnormal renal function is a common condition in patients with malignancy. Because LMWH is partially cleared by renal excretion and metabolism, drug accumulation is expected with significant renal insufficiency. Limited data are available on the use of LMWH in patients with significant renal dysfunction, but they do indicate that the risk of bleeding is higher in patients with renal impairment [95]. Recently, the development of NOACs is a millestone achievement in the prevention and treatment of VTE [95]. The major limitation is the lack of specific antidotes to reverse the anticoagulant effect and the absence of readily available assays to measure the coagulant effect, which can be an issue when facing bleeding events or treatment failure [95]. To date, NOACs have not been rigorously evaluated in cancer patients. A recent randomized phase II trial of apixaban for the prevention of thromboembolism in patients with metastatic cancer showed that apixaban is safe and feasible to use as VTE prophylaxis for high-risk cancer patients receiving chemotherapy. However, no published clinical trials have specifically addressed the treatment of cancer-associated VTE using these direct inhibitors. Also, the American Society of Clinical Oncology (ASCO) guideline does not recommend the use of these new agents [95]. In brief, LMWH is recommended for both initial and long-term anticoagulation in cancer-associated thrombosis by major consensus guidelines [94],[95]. If LMWH is unavailable, the ASCO 2013 VTE Prevention and Treatment Guideline recommend the use of VKAs with a target INR of 2–3 as an acceptable alternative [87]. For the treatment of cancer-associated VTE patients with renal impairment, if anti-factor Xa monitoring is not readily available, VKAs therapy is likely a safer option for long-term anticoagulation in these patients [95].

Regarding the proband (II-2), the presence of kidney cancer from this patient was detected at an early stage during the treatment with warfarin (1mg/day) for DVT of the leg at age 42 (via the observation of blood in urine (hematuria) (see case history report). In this case, the increased risk of warfarin-associated bleeding at the tumor site (when the tumor grows to a certain size, it will be under the pressure of the surrounding tissue that can lead to the burst of blood vessels on the walls of the tumor) was positively contributed to the early detection of kidney cancer from this patient [96]. Otherwise, the sequencing analysis of the tumor suppressor protein p53 (*TP53*) (GenBank X54156) and the human homologue of the murine double minute 2 protein (*HDM2*) (GenBank NC_000012.11) genes from genomic DNA isolated from whole peripheral blood of the proband (II-2) revealed no mutations (data not shown) [16]. These findings discard the presence of cancer due to mutations in *TP53* and *HDM2* genes from this patient. It is important to note herein that *TP53* gene is the most frequently mutated gene (>50%) in human cancer, indicating that the *TP53* gene plays a crucial role in preventing cancer formation [97],[98]. HDM2 protein is an important negative regulator of the p53 tumor suppressor protein. HDM2 protein functions both as an E3 ubiquitin ligase that recognizes the N-terminal trans-activation domain (TAD) of the p53 tumor suppressor protein and is an inhibitor of p53 transcriptional activation [99]–[101]. Inhibitors of the p53-HDM2 interaction might be attractive new anticancer agents that could be used to activate wild-type p53 in tumors. Down regulation of HDM2 using a small interfering RNA (siRNA) approach has recently provided evidence for a new role of HDM2 in the p53 response, by modulating the inhibition of the cyclin-dependent kinase 2 (cdk2) by P21/WAF1 (also known as cyclin-dependent kinase inhibitor 1 or CDK-interacting protein 1) [101].

Otherwise, it was reported that there is a cancer-associated arginine (R)-to-histidine (H) mutations confer a gain in pH sensing to mutant proteins [102], in which p53-R273H and the mutant epidermal growth factor receptor (EGFR-R776H) were subjected to this study [102]. The data show that Arg>His mutations can confer a gain in pH sensing to mutant proteins that is not seen with wild-type proteins, and suggest that Arg>His substitutions may provide a fitness advantage to the increased pH intracellular (pHi) of cancer cells. Moreover, lowering pHi attenuates some of the oncogenic effects of EGFR-R776H and partially restores p53-R273H tumor suppressor functions. Increased pHi is an established feature of most cancer regardless of tissue of origin or genetic background [102]. This increased pHi can enable tumorigenic properties, such as increased proliferation, cell survival, and metastasis. Increased pHi may both a cause and a consequence of tumor cell evolution. Whereas the evolutionary theory of cancer has largely been shaped by genomic analysis of tumor samples, cancer cell adaptation is mediated not by nucleotide changes but by proteomic changes that alter cell biology and enable cancer cell behaviors. Determining how distinct amino acid mutational signatures contribute to the physiological changes seen in cancer evolution is an important area of recent research. Recent wok has analyzed cancers by amino acid substitution signatures and found that Arg>His mutations are dominant in a subset of cancers but the physiological implications of this Arg>His amino acid mutation signature has not been determined or proposed. This work provides the first analysis of potential physiological relevance of the higher than expected frequency of Arg>His mutations observed in cancer. With a gain in pH sensing, Arg>His substitutions could provide an adaptive advantage to cancer cells by altering protein binding or activity specifically at increased pHi. These findings suggest that the tumorigenic effects of some somatic Arg>His cancer mutations become penetrant only at high pHi and suggest that lowering pHi in cancer cells may reduce the deleterious effects of some Agr>His mutations. This increased pHi can work in concert with mutant proteins to enhance oncogenic signaling and limit tumor suppression. These findings add to an emerging list of tumorigenic behaviors enabled by the established higher pHi of cancer cells and lay the groundwork for future studies on the functional effects of other amino acid substitutions that may allow adaptive and advantageous responses to either altered pHi dynamics or dynamic microenvironment pressures in cancers, such as oxidative stress, oxygen and nutrient availability, and metabolic reprogramming [102].

The use of conventional cancer chemotherapeutic agents are usually cytotoxic drugs which when successful, have a greater toxic effect on the tumor than on the host. Immuno-therapy and anticoagulant therapy are aimed at enhancing host response to the tumor [93]. Cancer patients are at increased risk of recurrent VTE and anticoagulant-associated bleeding [94],[95]. Thus, the management of VTE may be complex in patients with cancer, and VTE can further compromise quality of life. Because of the links between coagulation, cancer biology and prognosis, interest has growth in the potential benefits of anticoagulants such as warfarin and LMWH for the prevention or treatment of cancer [94],[95]. Warfarin is likely a safer option for long-term anticoagulation in patients with renal impairment [95]. Then, apart from warfarin anticoagulant, no specific treatment for cancer such as chemotherapy, radiotherapy, etc. was applied to the proband (II-2). Since 27 years, under low-dose of warfarin treatment (actually 3mg/day), and currently, he is well, free of cancer and thrombotic event. It appears that low-dose of warfarin is an effective and safe treatment in preventing VTE and cancer from this patient. This observation supports the “two ways association” idea of the relationship between cancer and thrombosis mentioned above [79]–[85]. That is, coagulation activation itself may contribute to the progression of some tumors and that anticoagulants may, therefore, have anti-cancer activity [86],[88]. It is evident that the “two ways association” between VTE and cancer cannot be substantiated by a single case description but this is the first report of a genetic trait that is shown as an example for the demonstration of such a relationship. Although some studies have suggested that warfarin may also improve survival in cancer patients and reduce the incidence of cancer [103]–[105], larger case-control studies are required to confirm the findings of this preliminary observation and more research is needed to further define which cancer type and stage would most benefit from warfarin. Otherwise, apart warfarin (3mg/day), the proband (II-2) is also under treatment with atorvastatin of 10mg/day (see case history report) to prevent high levels of cholesterol [106],[107] as well as atherosclerosis [108].

#### 3.2.2. Hypoxanthine-guanine phosphoribosyltransferase (HGprt) enzyme in Lesch-Nyhan disease (LND), thrombosis, and cancer

Lesch-Nyhan disease (LND) is a rare X-linked inherited neurogenetic disorder of purine metabolism affecting 1 in 380,000 people, and caused by deficiency of the soluble cytoplasmic hypoxanthine-guanine phosphoribosyltransferase (HGprt) enzyme (EC. 2.4.2.8; MIM 300800). This enzyme plays a central role in the generation of purine nucleotides from degraded DNA through the purine salvage pathways [109],[110]. LND is characterized by hyperuricemia, gout, nephrolithiasis, tophi, motor disability, intellectual impairment, and self-injurious behaviors such as self-biting, self-hitting, eye poking, and others. Complete or severe deficiency of HGprt activity leads to LND (MIM 300322). Self-injurious behavior is universal in LND. All information regarding the housekeeping hypoxanthine phosphoribosyltransferase 1 (*HPRT1*) gene that encodes the soluble cytoplasmic (HGprt) enzyme as well as the pathological conditions associated with the deficiency of HGprt activity found in LND and its variants: Lesch-Nyhan variants, LNVs, due to a partial deficiency of HGprt enzyme activity are described in [109]–[115]. These LNVs patients are characterized by consequences of overproduction of uric acid and variable spectrum of neurological manifestations, without the self-injurious behaviors [109]–[115]. How the loss of HGprt enzyme function affects the brain to cause the neurobehavioral syndrome in LND/LNVs, especially the self-injury of LND? For such a question, up to present, there is no valid answer. This has made difficult for the treatment development and has led to the absence of effective LND treatments [109]–[115]. Indeed, histopathological studies of autopsy tissues from LND patients revealed no signs suggestive of a degenerative process in any brain region [116]. On the other hand, and at the biochemical level, there was strong evidence that the neurological impairments in LND/LNVs were due to the effect of HGprt deficiency on the neural development, mainly, but not only, related to dopaminergic pathways [117],[118]. Nevertheless, none of these studies showed the pathogenic mechanism whereby HGprt deficiency affects the neuronal development, and the mechanism by which features of LND/LNVs result from impaired purine metabolism is still not well understood. However, it was also documented that:

- adhesion of HGprt-deficient neuroblastomas as well fibroblasts from patients with LND/LNVs exhibited dramatically enhanced adhesion compared to control [119], and could have consequences for the maturation of the central nervous system, as seen in the small brain size of LND/LNVs children [120]–[122];

**-** Alzheimer's disease (AD) shares gene expression aberrations with purinergic dysregulation of HGprt deficiency [123];

- role for the β-amyloid precursor protein (APP) is a key developmental gene related to cell-cell or cell-substrate adhesion, generation of neurons, their differentiation and migration, neurite outgrowth, regulation of synaptic function, and is important for brain morphology and highly coordinated brain function such as memory and learning has been suggested [124],[125].

**Figure 5.**
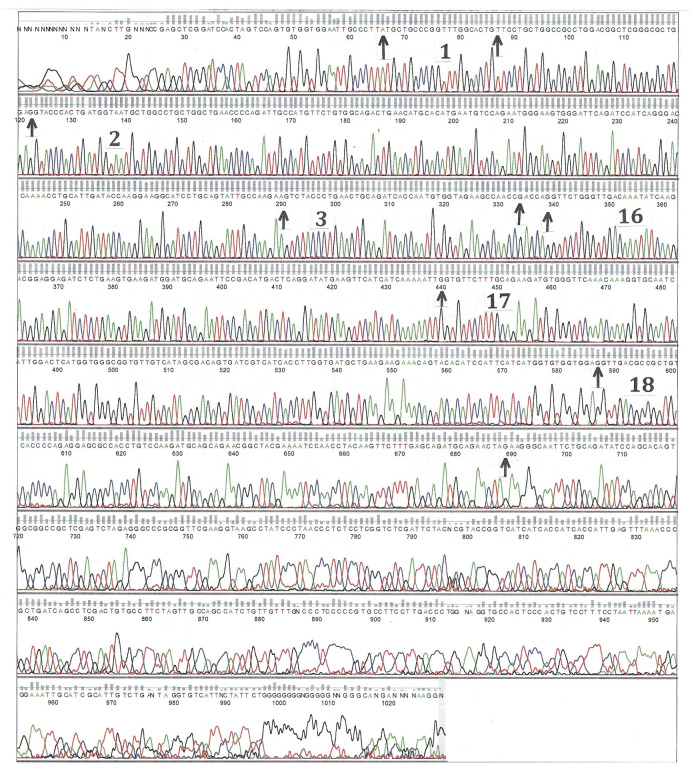
Chromatogram of the entire coding sequence (CDS) analysis of the APP-mRNA isoform of 624 bp encoding APP_207_ isoform obtained by RT-PCR coupled with direct sequencing from the cultured fibroblasts of a LND affected male patient #2 resulting from an IVS7 + 1G > A, c.532 +1G > A splice site mutation (see Tables 1 and 2 of Ref. #128). Based on GenBank NM_000484 with +1 as A of the ATG start codon, the CDS sequence read from left to right (5′ → 3′) in which the numbers #1–18 starting at A_66_ (↑) of initiation codon ATG for exon 1 and ending at G_689_ (↑), that is, Amber termination codon TAG for exon 18 of the CDS sequence of APP-mRNA isoform of 624 bp, showed a complete deletion of exons 4-15 starting after 49 bp of the 5′ end of exon 3, and mutations in exon 1: c.22C > T, p.L8F (see the presence of nucleotide T at bp 87: T_87_ in exon 1 of the chromatogram (↑)), and exon 3: c.269A > G, p.Q90R (see the presence of nucleotide G at bp 334: G_334_ in exon 3 of the chromatogram (↑)) encoding APP_207_ isoform. Protein mutation numbering is based on GenBank NP-000475.

Recently, however, it was demonstrated, for the first time, that expression of *APP* gene is under epigenetic regulation resulting in the presence of several APP messenger (APP-mRNA) isoforms encoding diver APP protein isoforms ranging from 120 to 770 amino acids (with or without mutations and/or deletions), and APP-mRNA isoforms with a deletion followed by an insertion (INDELS) accounted for epigenetic mechanisms in the regulation of alternative APP pre-mRNA splicing due to epigenetic modifications and/or epistasis (gene-gene interactions) as well as to epigenetic control of genomic rearrangements of *APP* gene (Table 2) [126],[127]. In addition, a report on the quantification of various APP-mRNA isoforms in biological samples, especially for identifying the most abundant one that may decisive for the normal status or disease risk has been described and applied for identifying the defective APP-mRNA isoform in LND. The results indicated, for the first time, a role for epistasis between mutated *HPRT1* and *APP* genes affecting the regulation of alternative APP pre-mRNA splicing (APP-mRNA isoform of 624 bp, with a deletion starting after 49 bp of the 5′ end of exon 3 followed by a complete deletion of exons 4–15, mutations in exon 1: c.22C > T, p.L8F, and exon 3: c.269A > G, p.Q90R encoding APP_207_ isoform, was the most abundant one in most of the LND patients and would be responsible for the neurobehavioral syndrome in these patients) (Figure 5) [128]. Furthermore, there were also some reported cases of LND/LNVs developing thrombosis [129]–[131] while APP is an important regulator of vein thrombosis and controls coagulation and neutrophil extracellular traps (NETs) formation via the Kunitz-type serine protease inhibitor (KPI)-containing the α soluble fragment of APP (APPsα fragment) that were demonstrated in vitro to be effective inhibitors of the coagulation FXa, FIXa, FXIa, and FVIIa:tissue factor complex [132]. Then, APP pathway could be implicated in the development of neurological features as well as thrombotic events of LND/LNVs. Otherwise, the surface expression of HGprt enzyme was also observed in several somatic tissue cancers [133]–[139] while an important function of APP as a tumor growth factor in the pathogenesis of several somatic tissue cancers has been suggested and APP as well as APP-like protein-2 (APLP2) are deregulated in cancer cells and linked to increase tumor cell proliferation, migration, and invasion [140],[141]. These findings suggest an emerging role of HGprt in cancer development.

**Table 2.** Isoforms of APP and mutations/deletions/insertions.

Samples^a^	Isoforms	Mutations and/or Deletions
1	APP_770_	No mutation
	APP_770_	Mutation in exon 5: c.622T>C, p.V208A
	APP_203_	Deletion starting after 102 bp of the 5′ end of exon 5 followed by a complete deletion of exons 6-16, and 104 bp of the 5′ end of exon 17. Mutation in exon 2: c.135A>G, p.N46D
	APP_168_	Deletion starting after 93 bp of the 5′ end of exon 3 followed by a complete deletions of exons 4-16, and 59 bp of the 5′ end of exon 17. No mutation
7	APP_770_	Mutations in exon 6: c.751G>A, p.G251D; exon 7: c.979A>G, p.N327S
	APP_770_	Mutations in exon 10: c.1249A>G, p.E417G; exon 11: c.1429T>C, p.I477T; exon 13: c.1657C>T, p.A553V
	APP_207_	Deletion starting after 49 bp of the 5′ end of exon 3 followed by a complete deletion of exons 4-15. Mutations in exon 1: c.21C>T, p.L8F; exon 3: c.268A>G, p.Q90R
	APP_120_	Deletion starting after 27 bp of the 5′ end of exon 3 followed by a complete deletion of exons 4-16, and 138 bp of the 5′ end of exon 17. No mutation
	APP isoform with INDELS: c.19_2295delinsG_166_TT...GAGTCC...CTTAG TC...TCT_489_, p.Leu7Valfs*2 in which there was a deletion followed by an insertion of 324 bp between exon1 and exon 18 of *APP* gene resulted from an interchromosomal rearrangements between APP (located on chromosome 21q21.2-3) and erythrocyte membrane protein band 4.1-like 2 (EPB41L2, located on chromosome 6q23.1-q23.2) loci (the underlined letters T, A, and A, G indicate the difference in nucleotides in the sequence of 324 nucleotides inserted for samples # 7 and # 15).	
13	APP_770_	No mutation
	APP_770_	Mutation in exon 12: c.1563delA, p.K522fs531X in exon 13
	APP_751_	Mutation in exon 15: c.1930C>T, p.P644L
	APP_751_	Mutations in exon 12: c.1557C>T, p.P520S; c.1570C>T, p.A524V; exon 16: c.2062T>C, p.L688S
	APP_216_	Deletion starting after 33 bp of the 5′ end of exon 3 followed by a complete deletion of exons 4-14, and 11 bp of the 5′ end of exon 15. No mutation
	APP_168_	Deletion starting after 63 bp of the 5′ end of exon 3 followed by a complete deletion of exons 4-16, and 30 bp of the 5′ end of exon 17. No mutation
14	APP_770_	No mutation
	APP_770_	Mutation in exon 2: c.135A>G, p.N46D
	APP_334_	Deletion starting after 9 bp of the 5′ end of exon 6 followed by a complete deletion of exons 7-15, and 15 bp of the 5′ end of exon 16. No mutation
	APP_193_	Deletion starting after 42 bp of the 5′ end of exon 3 followed by a deletion of 209 bp of the 5′ end of exon 14. Complete deletion of exons 4-13. Mutation in exon 2: c.199delC, p.Q74fs86X in exon 3. Mutation in exon 3: exon 3: c.242G>T, p.Q81H.
	APP_175_	Deletion starting after 132 bp of the 5′ end of exon 2 followed by a complete deletion of exons 3-15, and 10 bp of the 5′ end of exon 16. Mutation in exon 18: c.2265G>A, p.G756S
	APP isoform with INDELS: c.16_2313delinsG_84_CC...CAT_616_, p.Leu7Hisfs*45 in which there was a deletion followed by an insertion of 533 bp in exon 1 of *APP* gene resulted from an interchromosomal rearrangements between APP (located on chromosome 21q21.2-3) and the phosphogluconate dehydrogenase (PGD, located on chromosome 1p36.22) loci.	
15	APP_770_	No mutation
	APP_770_	Mutations in exon 9: c.1215A>G, p.M406V; exon 10: c.1380T>A, p.D427E; exon 16: c.2050A>G, p.H684R
	APP isoform with INDELS: c.19_2295delinsG_169_TT...GAGACC...CTTGG TC...TCT_492_, p.Leu7Valfs*2 in which there was a deletion followed by an insertion of 324 bp between exon1 and exon 18 of APP gene resulted from an interchromosomal rearrangements between APP (located on chromosome 21q21.2-3) and erythrocyte membrane protein band 4.1-like 2 (EPB41L2, located on chromosome 6q23.1-q23.2) loci (the underlined letters T, A, and A, G indicate the difference in nucleotides in the sequence of 324 ucleotides inserted for samples # 7 and # 15).	

^a^Samples used are: sample # 1 is normal subject, control; samples # 7,13 are LND affected male patients; samples #14,15 are LNV affected male patients.

##### 3.2.2.1. APP-HGprt and thrombosis in LND/LNVs

The findings as described in [129]–[131] support the impact of APP on LND/LNVs and suggest a potential molecular link between APP and HGprt enzyme via epistasis between mutated *HPRT1* and *APP* genes. Indeed, (1) the KPI domain, which is located in exon 7: amino acid residues 291–341 of the extracellular domain of APP_770_ and APP_751_ isoforms [142],[143], has been shown *in vitro* to be a potent inhibitor of the coagulation FXa, FIXa, FXIa, and FVIIa: tissue factor complex [132],[144],[145]. Furthermore, in addition to brain, APP is also expressed in extraneuronal tissues, mostly in platelets in which the APP_751_ and APP_770_ isoforms are expressed [132]. On platelet activation and under physiologic conditions, the majority of APP is processed via the non-amyloidogenic pathway via α-secretase, which is activated by a Ca^2+^/calmodulin-dependent mechanism [132],[142],[143]. Then, the processing of APP from platelets releases the KPI-containing soluble APPsα fragments [142],[143] that are analogous to protease nexin-2 (PN-2) [132],[144],[145]. PN-2/APP and its KPI domain have been demonstrated *in vitro* to be potent inhibitors of trypsin, chymotrypsin, epidermal growth factor binding protein, the γ subunit of nerve growth factor, and several key prothrombotic proteinases including factor XIa, factor IXa, factor Xa, and tissue factor: factor VIIa complex [132],[144],[145]. *In vivo* studies on APP/KPI^R13I^ mutant mice (mutation of the center basic arginine 13 residue of the KPI domain to the similar sized, but hydrophobic, isoleucine) [145], and APP-knock out mice have indicated that, as a result, APP negatively controls thrombosis [132]. It is important to note herein that (a) APP and APLP2 (possesses a highly conserved KPI domain that is highly homologous with the one contained in APP) are expressed ubiquitously throughout the body, mostly abundant in the nervous system; whereas APP-like protein-1 (APLP1) does not contain a KPI domain, is predominantly expressed in the nervous system [142],[143]. Here, similar to APP, APLP2 has been also shown *in vitro* and *in vivo* to have inhibitory activity against these hemostatic enzymes factors and regulates thrombosis [144]. These findings demonstrate an important role for platelet APP and APLP2 (expressed at a lower level) are both proteolytic inhibitors, through the KPI activity of the protein, that possess overlapping and shared activities contributing to the regulation of blood clot formation, limiting thromboembolic diseases as well as cerebral venous thrombosis [132],[144],[145]; (b) the severity of the prothrombotic risk of APP and, more recently, APLP2 have been proposed as cerebral anticoagulants [144]; (2) as previously mentioned, expression of APP gene is under epigenetic regulation resulting in the presence of several APP-mRNA isoforms (with or without mutations and/or deletions), encoding diver APP protein isoforms accounted for epigenetic mechanisms in the regulation of alternative APP pre-mRNA splicing was reported [126],[127]. In addition, a report on the quantification of various APP-mRNA isoforms in biological samples, especially for identifying the most abundant one that may be decisive for the normal status or disease risk has been described and applied for identifying the defective APP-mRNA isoform in LND [128].

Taking into consideration these findings, a miss regulation of alternative APP pre-mRNA splicing could lead to the presence of the most abundant APP-mRNA isoform that would be a defective one encoding consequently a defective APP protein isoform (or its proteolytic fragments) with mutation and/or deletion in the KPI domain such as the APP/KPI^R13I^ [145] or the 624 bp of APP-mRNA isoform encoding APP_207_ isoform as described in [128]. These defective isoforms could affect anticoagulant functions and abolishes therefore their anti-thrombotic activity. In this case, the overlapping compensatory effect of APLP2 would be decisive for the preservation of anti-thrombotic activity. This could explain the development of thrombosis from some LND/LNVs patients as described in [129]–[131].

##### 3.2.2.2. APP-HGprt and cancer

Salvage enzyme, such as HGprt and is known as a housekeeping protein, in which its important role is responsible for the production of nucleotides such as GTP and ATP that are necessary to providing energy for several cellular process as well as for regulating cell proliferation [109],[110]. Expression of *HPRT1* is cytosolic within all normal cells and maintained stable and low levels in normal tissue. Here, some questions remain to be elucidated such as (a) How HGprt is able to localize to the surface?; (b) Does it provide any functional advantage to the cancer cell?; (c) Determine the reason some cells that express HGprt on the surface while others do not? The observed surface expression of HGprt on certain malignancies makes it promising as a biomarker in the early diagnosis of cancer such as lung and colorectal cancer [133],[134],[136]. Surface expression of colorectal cancer cells has been also observed for the vitamin D3 receptor, and serves as a maker for such a cancer [136]. It is important to note that APP is ubiquitously expressed in a broad spectrum of cell types including non-neuronal cells, and it is suggested to be involved in the growth of these cells [142],[143],[146], while the nature of APP has been mainly studied in neuronal cells due to its pathological significance in AD. Recently, increasing evidence suggests an important function of APP as a potent tumor growth factor in the pathogenesis of several somatic tissue cancers, and APP as well as APLP2 are deregulated in cancer cells and linked to increased tumor cell proliferation, migration, and invasion [140],[141]. These findings suggest a potential link between APP and HGprt in cancer development. Indeed, it was demonstrated that expression of *APP* gene is under epigenetic regulation and a role for epistasis between *HPRT1* and *APP* genes affecting the regulation of alternative APP pre-mRNA splicing was also suggested [126]–[128]. A miss regulation could lead to the presence of the most abundant APP-mRNA isoform that would be a defective one encoding consequently a defective APP protein isoform (or its proteolytic fragments) capable of promoting cancer growth. In cancer, cells rapidly divide, the need for nucleotides increases, and as a result HGprt is upregulated and some cancer cells express HGprt on the surface for the purpose of inducing changes in the metabolism and activity to maintain rapid tumor cell proliferation.

In summary, the examples discussed here suggest strongly a potential molecular link between APP and HGprt via epistasis between *HPRT1* and *APP* genes, and highlight the impact of alternative splicing (AS) process on human disease, and clearly show that how AS is dynamically regulated and generates isoform diversity with critical functions and a misregulation of AS plays a large role in numerous human diseases. An accurate quantification of various APP-mRNA isoforms from different tissues for identification the most abundant APP-mRNA isoform that may be decisive for the normal status or disease risk is needed and antisense drugs are the potential treatments [128],[147]. As a perspective, for clarification of these issues, it is necessary to study the HGprt enzyme and APP using expression vectors for exploring their impacts on LND as well as other human diseases, especially the ones related to APP such as AD and cancer [141]. For such a purpose, the construction of expression vectors for HGprt and APP was performed [148]. These expression vectors, with or without the glycosyl-phosphatidylinositol (GPI) anchor, could be used as tools for (a) studying the effects of mutation on HGprt enzyme found from different LND/LNVs patients; (b) studying the emerging role of *HPRT1* gene in cancer, especially exploring the effects for the surface expression of *HPRT1* gene; (c) exploring the mechanism linking HGprt deficiency, purinergic pathways, and neural dysfunction of LND; (d) exploring the structure and the physiologic function of APP; (e) studying intermolecular interactions between APP and HGprt enzyme.

## 4. Conclusion

VTE is now considered to be a chronic disease, in that the risk for recurrent persist for many years after the initial event. This is the first report of inherited AT deficiency: Arg393His in AT-Hanoi characterized by CVT (encephalomalacia), DVT, and kidney cancer found from the proband (II-2) [16] (see case history). The use of PCR coupled with direct sequencing performed from DNA isolated from buccal cells in mouthwash allowed the molecular characterization of AT deficiencies in the simplest manner with no need of blood collection from patients. It is anticipated that a better understanding of the interactions between tumor growth and blood coagulation, together with the results of the clot trial, will help to improve the prophylactic and treatment strategies for VTE. A question arises from this study concerns the highly variable from asymptomatic to severe recurrent VTE or arterial thrombosis, leading to death [149],[150]. In addition, the age of onset of the first thrombotic episode exhibited by a patient with hereditary AT deficiency varies considerably [151]. There are probably other yet undiscovered factors other than the genotype at the AT locus such as modifier genes and environmental factors acting in the phenotypical expression of the disease associated with AT deficiency. This issue concerns then epigenetic modifications in epistasis and/or gene-environment interactions [152]–[154]. Another question arises as to whether asymptomatic patients with no additional risk factors should undergo some prophylactic therapy given that long-term anticoagulation carries with it significant risks such as bleeding [61],[62].

In recent years, there have been significant advances in our understanding of the molecular mechanisms associated with increased risk of VTE in cancer such as aPL presence associated with malignancies and thrombotic event [91], cancer-associated Arg>His mutation [102], although there remain significant gaps in our knowledge of the causes of and best approaches for thromboprophylaxis in cancer-associated thrombosis. More research in this field should lead to a better understanding of the pathophysiology and optimal therapeutic approaches for the prevention of cancer-related thrombosis. Furthermore, through a massive research effort over the last two decades focusing on epigenetic modifications in epistasis and/or gene-environment interactions as well as attempts to characterize the impacts of APP on thrombosis and cancer related to LND [109]–[147] are likely to emerge in the near future and will help to improve the prophylactic and treatment strategies for LND, VTE as well as cancer.

## 5. Perspective

**Figure 6.**
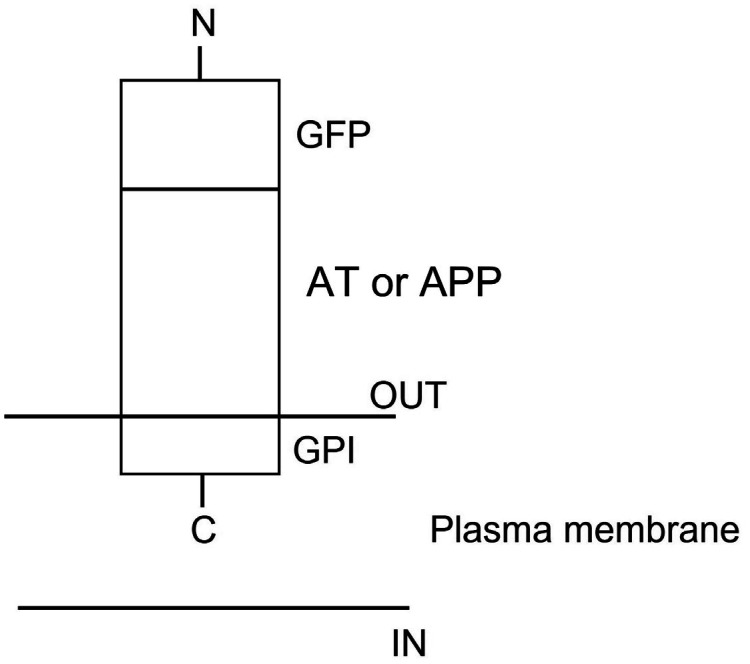
Schematic representation of the membrane topology of the expression vectors for human AT and APP. The construct comprising the sequence encoding the C-terminal of the glycosyl-phosphatidylinositol, GPI, anchor derived from the human folate receptor (FOLR1) protein; the entire coding sequence (CDS) of *AT* or *APP* gene coupled with the CDS of the green fluorescence protein (*GFP*) gene.

Epistasis is important, ubiquitous, and has become a hot topic in complex disease genetics such as AD, schizophrenia, autism, cancer, etc. in recent years. A gene does not function in isolation and by itself, but rather acts with other genes in a network, to influence complex traits of the complex disorders. However, the data supporting epistasis in complex human diseases are emerging slowly. This is due to different difficulties that we face in detecting and characterizing epistasis, such as challenges of modeling non-linear interactions, and in the interpretation of results [128],[152]–[154]. APP, a housekeeping gene and an endogenous ligand (http://www.genenames.org/genefamilies/ENDOLIG) [128],[142],[143], is an important molecular hub at the center of interacting pathways and acts as a permissive factor for various cellular functions, and therefore it is not surprising that altered APP processing may affect neuronal as well as non-neuronal cellular functions through a host of altered cellular and molecular events found in human diseases. Furthermore, α-, β- and γ-secretase processing of APP (at the N-and C-terminals of the Aβ sequence) also occur under physiological conditions; this indicates that all fragments of APP, including the Aβ peptide, are part of normal physiology [142],[143]. The targeting of the components of APP processing as a pharmacologic strategy will not be without consequences. Therefore, it is important to continue to investigate the normal function of APP. Understanding its physiological function will not only provide insights into the pathogenesis of diseases but may also prove vital in the development of an effective therapy. The role of epigenetics in rare diseases is a key issue in molecular physiology and medicine because the understanding about the mechanisms that explain the influences of epigenetic regulation in rare diseases will provide useful principles for other common and complex disorders. Epigenetic regulation determines not only what parts of the genome are expressed but also how they are spliced [141]. The examples discussed here highlight the impact of alternative splicing (AS) process on human disease, and clearly show that how AS is dynamically regulated and generates isoform diversity with critical functions and a misregulation of AS plays a large role in numerous human diseases. An accurate quantification of various APP-mRNA isoforms from different tissues for identification the most abundant APP-mRNA isoform that may decisive for the normal status or disease risk is needed and antisense drugs are the potential treatments [128],[147]. It is therefore necessary to study the HGprt enzyme, AT, and APP using expression vectors for exploring their impact on LND, thrombosis as well as other human diseases, especially the ones related to APP such as AD and cancer [141]. For such a purpose, the construction of expression vectors for HGprt and APP was performed [148]. In the same manner, the construction of expression vectors for AT and APP can be performed as shown in Figure 6. These expressions vectors, with or without GPI anchor, could be used as tools for (a) studying the effects of Arg393His mutation in AT; (b) studying the emerging role of Arg393His mutation in AT and cancer; (c) studying intermolecular interactions between APP and AT.

It is also important to note herein that the construction of expression vectors as described in [148], especially the one with GPI can be used as a model for the construction of expression vectors for any protein targeting to the cell plasma membrane for studying intermolecular interactions and could be therefore useful in the vaccines as well as antiviral drugs development (studying intermolecular interactions between the spike glycoprotein of the severe acute respiratory syndrome coronavirus 2, SARS-CoV-2, as well as its variants and the angiotensin-converting enzyme 2, ACE2, in coronavirus disease 2019 (COVID-19) [155],[156], for example).
